# The pharmacist’s role in managing and ameliorating childhood’s asthma care: a descriptive and transversal study about 104 pharmacists in the city of Rabat in Morocco

**DOI:** 10.11604/pamj.2018.30.101.11593

**Published:** 2018-06-06

**Authors:** Bousayna Iraqi, Imane Jroundi, Amina Iraqi, Chafiq Mahraoui

**Affiliations:** 1 Pediatric Pulmonary and Allergic Diseases Unit, Ibn Sina Children’s University Hospital, Rabat, Morocco; 2 Unit of Research and Training of Public Health, School of Medicine and Pharmacy University Mohammed V, Rabat, Morocco; 3 King Fahd School of Translation-Tangier, Morocco

**Keywords:** Pharmacist, childhood asthma, therapeutic education, treatment, Morocco

## Abstract

This work aims at enhancing the management of childhood asthma, with a focus on pharmacists in particular, by evaluating their knowledge of childhood asthma and assessing their attitude while they are providing asthma medicines. Consequently, it will look at the necessity of introducing training days about childhood asthma for pharmacists. This is a transversal and descriptive study which lingered from August to October 2015. Data has been collected using a questionnaire that was self-administered to every surveyed pharmacist in the city of Rabat. The 104 pharmacists who replied to the questionnaire have an average general understanding about asthma and its treatment. Only a quarter of them managed an asthma crisis in their pharmacy before directing the child to the emergency. 50% of them do not know the difference between the basic asthma therapy and the asthma attack therapy. However, all of them recommended the parents to see a physician regarding their child’s asthma. 75% advise the systematic use of an asthma spacer with the inhaler. 87.5% of them give advice to parents regarding the good measures for environmental control, and 98% estimate that the therapeutic education is important in childhood asthma management. 88.5% among them are interested in training days about childhood asthma.This study shows the necessity of further pharmacists’ education about asthma and its management.

## To the editors of the Pan African Medical Journal

Asthma is the most frequent chronic disease in Morocco, affecting 20% of Moroccan children [1]. Asthma treatment involves two difficulties, namely the non-compliance with the prescribed treatment and the bad use of the inhalation devices [2]. The management of childhood asthma is not limited to consultations with the pneumo-pediatrician. The pharmacist should play a complementary role to that of the physician when it comes to the therapeutic education of asthma [3]. The pharmacist is also supposed to play an essential role in helping in the diagnosis because, in certain situations, he could be the first interlocutor to whom the parents of an asthmatic child would describe the symptoms of their child. This work aims at assessing the knowledge and attitude of pharmacists while they are providing asthma medicines for childhood asthma.

This is a transversal descriptive study conducted among pharmacists working in private offices in the city of Rabat, which lingered from August to October 2015. Data has been collected using a standardized questionnaire that was self-administered to every surveyed pharmacist. The study has included 50% of dispensary pharmacists extracted from the official list of the pharmacists who are practicing in the city of Rabat (n = 268). The extraction was done using a random draw method from a random list of numbers. An explanatory meeting with every pharmacist has been held for the sake of a better understanding and collaboration. A sample of 134 pharmacists was randomly selected. The rate of answers to the questionnaire was 77.6% (n = 104). The gender ratio (F/M) was 0.46. A percentage of 56% (n = 58) of interviewed pharmacists had between 10 to 30 years of experience.

More than 52% of the interviewed pharmacists think that Asthma is not a chronic disease. 90% confirm that asthma is a lung allergy that could be healed by avoiding triggering factors. 90% are aware that asthma could lead to death. A percentage of 14.4% of interviewed pharmacists estimate that an asthmatic child should not practice a sport activity, whereas 86.7% estimate that allergic rhinitis is an aggravating factor of child asthma. Regarding the assessment of the Knowledge and behavior during an asthma crisis, a percentage of 55.3% of the pharmacists require a prescription before issuing treatment to the asthmatic child,and all of them recommended the parents to see a physician regarding their child’s asthma. However, only a quarter managed an asthma crisis in their pharmacy before directing the child to the emergency. From the 104 interviewed pharmacists, 36% were frequently giving oral corticoids to an asthmatic child while 57.7% refused to deliver oral corticoids without a medical prescription. The degree of the estimated medicines’ utility by pharmacists is represented in Table 1. Half of our pharmacists do not know the difference between the basic asthma therapy and the asthma attack therapy. However, 63.5% estimate that the inhaled corticotherapy is efficient during asthma attacks. A percentage of 75% of pharmacists advise the systematic use of an asthma spacer with the inhaler by an asthmatic child. 90.4% among them explain the use of the chamber in every issued prescription, and 73% among them verify the good use of the inhalation device in every issued prescription. The majority (87.5%) of the interviewed pharmacists give advice to patients regarding the good measures for environmental control. 98% of the interviewed pharmacists estimate that the therapeutic education is important in childhood asthma management. More than half of them (52%) find difficulties in answering all the questions asked by parents, and 88.5% among them would be interested in training days about childhood asthma.

**Table 1:** The medicines used during an asthma attack according to the interviewed pharmacists

	Used during the attack	Not used during the attack	Do not know
**Oral corticosteroids**	**82** **78,8%**	13 12,5%	9 8,7%
**Beta 2 mimetic syrup**	37 35,6%	35 33,7%	32 30,8%
**Beta2 mimetic spray**	**80** **63,5%**	9 8,7%	**15** **14,4%**
**Inhaled corticosteroids**	**66** **63,5%**	23 22,1%	15 14,4%
**Antileukotrienes**	28 26,9%	41 39,4%	35 33,7%
**Antitussives**	9 8,7%	**64** **61,5%**	31 29,8%
**Antibiotics**	12 11,5%	**61** **58,7%**	31 29%

**Figure 1:**
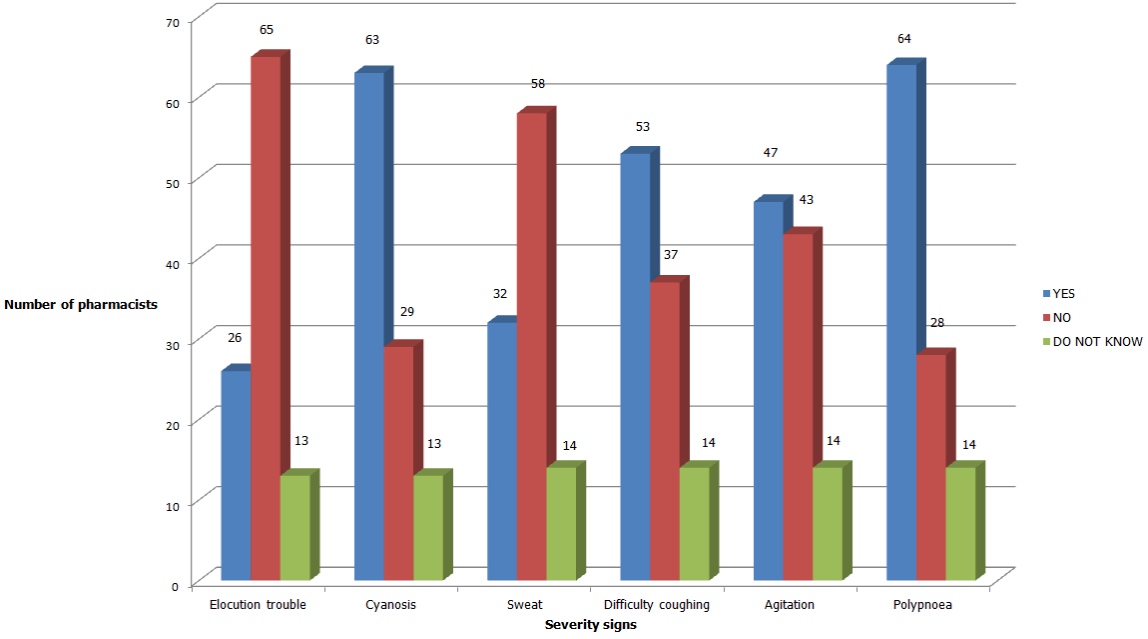
Asthma attack’s signs of severity as estimated by the interviewed pharmacists

Our results demonstrate that the interviewed pharmacists do only have an average general knowledge about asthma disease and its treatment. In fact, a non-negligible percentage of pharmacists estimated that an asthmatic child should not practice sport despite the fact that sport, and particularly swimming, is advised for asthmatic children because it increases their respiratory function and combatobesity which is considered as a comorbidity factor for childhood asthma [4, 5]. As far as the comorbidity factors are concerned, our pharmacists are aware that asthma treatment cannot be performed without the treatment of allergic rhinitis [6]. However, they all knew less about the seriousness of asthma symptoms (Figure 1), which would be an essential point in these pharmacists’ training about the asthma disease. Upon their reception of an asthma attack’s case, the majority of the interviewed pharmacists refused to administer the beta 2 agonist at the pharmacy and referred the children to the emergency. Most of them justify this refusal using the medical legal framework and the lack of training. These results are different from those of other countries [7, 8]. Additionally, according to the Moroccan deontology code which regulates the profession of pharmacists, the pharmacist shall rescue a patient which is in immediate danger if medical care could not be provided [9]. 75% of the interviewed pharmacists said that they advised on the use of an inhalation chamber with a measuring spray and confirmed explaining its use upon the delivery of every prescription. However, our investigation could not evaluate this point. The given responses could not reflect the reality of the current practices. This finding further surpasses that of the study conducted in Australia where only 50% of the pharmacists affirm demonstrating the manipulation of the inhalation chamber for asthmatic children [10]. The findings cannot be generalized to all the pharmacists of Morocco. Our study has focused on the pharmacists of the city of Rabat, an urban area, where the medical coverage is important. Another limitation of this study is that because the questionnaire was self-administered, we cannot confirm the absence of reliance on an external aid that could influence the collected answers.

## Conclusion

Our results show that pharmacists have an average knowledge about asthma and its management. The interviewed pharmacists lack knowledge about the severe criteria of asthma attacks; they also lack knowledge about the difference between asthma basic therapy and asthma attack therapy. The majority of them reported having found difficulties in answering the parents’ questions and expressed their interest in receiving training modules about asthma. This research shows the necessity of furthering pharmacists’ training in asthma and its therapeutic strategy as well as sensitizing them about the severity of asthma attacks signs. This study could also be used as a catalyst for the definition of the important points for discussion during the asthma training modules.

## Competing interests

The authors declare no competing interest.
